# Insights into Structures and Dynamics of Flavivirus Proteases from NMR Studies

**DOI:** 10.3390/ijms21072527

**Published:** 2020-04-05

**Authors:** Qingxin Li, CongBao Kang

**Affiliations:** 1 Guangdong Provincial Bioengineering Institute (Guangzhou Sugarcane Industry Research Institute), Guangdong Key Lab of Sugarcane Improvement & Biorefinery, Guangzhou 510316, China; 2 Experimental Drug Development Centre (EDDC), Agency for Science, Technology and Research (A*STAR), 10 Biopolis Road, Chromos, #05-01, Singapore 138670, Singapore

**Keywords:** flavivirus, Dengue virus, Zika virus, West Nile virus, protease, NMR, protein structure

## Abstract

Nuclear magnetic resonance (NMR) spectroscopy plays important roles in structural biology and drug discovery, as it is a powerful tool to understand protein structures, dynamics, and ligand binding under physiological conditions. The protease of flaviviruses is an attractive target for developing antivirals because it is essential for the maturation of viral proteins. High-resolution structures of the proteases in the absence and presence of ligands/inhibitors were determined using X-ray crystallography, providing structural information for rational drug design. Structural studies suggest that proteases from Dengue virus (DENV), West Nile virus (WNV), and Zika virus (ZIKV) exist in open and closed conformations. Solution NMR studies showed that the closed conformation is predominant in solution and should be utilized in structure-based drug design. Here, we reviewed solution NMR studies of the proteases from these viruses. The accumulated studies demonstrated that NMR spectroscopy provides additional information to understand conformational changes of these proteases in the absence and presence of substrates/inhibitors. In addition, NMR spectroscopy can be used for identifying fragment hits that can be further developed into potent protease inhibitors.

## 1. Introduction

Flavivirus is a genus of viruses in the family *Flaviviridae*, and some members such as Dengue virus (DENV), West Nile virus (WNV), Zika virus (ZIKV), tick-borne encephalitis virus, and yellow fever virus are important human pathogens [1,2,3]. Some of these viruses can be transmitted by mosquitoes. Although viral infection usually causes mild symptoms such as fever and joint pain, it can also cause serious diseases such as dengue fever, microcephaly in newborns, and Guillain–Barré syndrome in adults [4,5,6,7]. Chemical therapy and vaccines are needed to prevent viral infection. Developing small-molecule drugs targeting viral proteins or preventing virus–host interactions is a strategy to develop antivirals [8,9,10,11].

The genome of *Flaviviridae* is a single-stranded RNA which has one open reading frame encoding a single polyprotein [1]. The polyprotein is cleaved into three structural and seven non-structural (NS) proteins by both host and viral proteases [12]. The structural proteins including the capsid protein (C), membrane protein (M), and envelope protein (E) are important for forming viral particles and receptor binding [13]. The seven non-structural proteins include NS1, NS2A, NS2B, NS3, NS4A, NS4B, and NS5 which are important for viral replication [1]. The structures and functions of these proteins are well conserved across the family. Among these non-structural proteins, NS3 and NS5 possess enzymatic activities, making them attractively studied [14,15]. 

The flavivirus protease is a two-component complex formed by a cytoplasmic region of NS2B and approximately 170 amino acids from the N-terminus of NS3 [16,17,18]. NS2B is a membrane protein with four transmembrane helices and acts as a cofactor for NS3 by regulating its activity and membrane location [18]. The transmembrane domains of NS2B are critical for the membrane location of both NS2B and NS3 [17]. These domains might be also important for the membrane location of replication complex formed by the viral proteins. The protease cofactor region of NS2B comprises approximately 40 amino acids and is critical for folding and protease activity of NS3 by forming a tight complex with the N-terminal region of NS3, with involvement in molecular interactions with substrates [19,20]. The N-terminus of NS3 (NS3pro) contains amino acids critical for the cleavage of substrates. The flavivirus protease is normally referred to as NS2B–NS3 protease (NS2B–NS3pro) due to the presence of two proteins. The viral NS2B–NS3 protease cleaves the joints of NS2A–NS2B, NS2B–NS3, NS3–NS4A, and NS4B–NS5 proteins, which is essential for maturation of these viral proteins [1,2]. Due to the important roles of NS2B–NS3 protease, it is an important antiviral target [21,22]. 

Structural studies of a target protein and its complex with a ligand/substrate provide useful information for rational drug design [23]. X-ray crystallography is a powerful tool to elucidate structures of proteins and their complexes at a high resolution. Solution NMR spectroscopy, on the other hand, will provide additional information to understand protein structures and dynamics under physiological conditions [24,25,26,27]. In the structural studies of proteases from several members of flaviviruses, these two methods were used together to provide valuable information to understand protease structure, dynamics, and ligand-binding properties [28,29]. The viral protease is an attractive target as it is essential for maturation of non-structural proteins. Therefore, structural information is very useful for designing potent inhibitors. In this review, the roles of solution NMR spectroscopy in understanding the dynamics and structures of proteases from DENV, WNV, and ZIKV are discussed. Accumulated studies showed that NMR, together with X-ray crystallography, is critical in the development of protease inhibitors. 

## 2. X-ray Structures of Flavivirus Proteases

Numerous crystal structures of flavivirus proteases in the absence and presence of inhibitors were determined [30,31,32,33,34,35,36,37,38,39,40]. All these structures use recombinant proteins lacking the transmembrane domains of NS2B, as folding of full-length NS2B requires the presence of membrane systems such as detergent micelles [41,42,43,44]. For DENV and WNV proteases, a construct containing a cofactor region, approximately 40 residues from NS2B and NS3 protease domain (NS3pro) linked via a glycine-rich linker, was used in the structural studies [45]. In this manuscript, NS2B refers to the cofactor region, while full-length NS2B means the entire NS2B protein containing all the transmembrane domains. 

### 2.1. Structures of Proteases in Complex with Substrates/Inhibitors 

**Figure 1:**
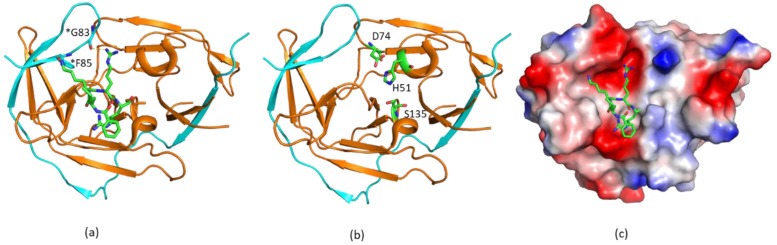
Structures of West Nile virus (WNV) protease in complex with an inhibitor. (**a**) The crystal structure of WNV NS2B-NS3pro in complex with an inhibitor. The non-structural protein 2B (NS2B) cofactor region and NS3pro are shown in cyan and orange, respectively. The inhibitor is shown in sticks, and residues from NS2B having close contacts with the inhibitor are labeled. Residues from NS2B are labeled with an asterisk. (**b**) The catalytic triad of WNV protease. The residues forming the catalytic triad are labeled and shown in sticks. (**c**) Surface representation of WNV protease. Surface charge analysis of the structure is shown. The structure was obtained from the Protein Data Bank (PDB) with access code 2FP7.

In structures of DENV, WNV, and ZIKV NS2B–NS3 proteases, the folds of NS3 in various X-ray structures are almost identical (Figure 1). The N-terminal domain of NS3 is a serine protease containing two β-barrels, and each barrel consists of six β-strands. The catalytic triad formed by His, Asp, and Ser residues is absolutely conserved among these proteases (Figure 1) [30]. The active site is negatively charged, favoring molecular interactions with positively charged residues such as Lys and Arg (Figure 1) [46]. As shown in one of the crystal structures of WNV NS2B–NS3pro in complex with an inhibitor, the S1 pocket of the protease comprises Asp 129, Tyr130, Thr132, Tyr 150, and Gly151. Asp129 stabilizes the positively charged side chain of P1 residue, serving a similar function to Asp189 in trypsin [47]. The S2 pocket is negatively charged by the contribution of electrostatic potential from the backbone carbonyl oxygen atoms of amino acids of NS2B. Asp152 of NS3 and Gly83 of NS2B might form hydrogen bonds with the positively charged group of P2 residue (Figure 1a). The cofactor region of NS2B adopts two different conformations in some crystal structures (see below). In the absence of NS3pro, the free cofactor region of NS2B is unstructured in solution. The N-terminal part of the cofactor region forms a stable complex with NS3 and exists as a β-strand (Figure 1a). This region itself is able to stabilize NS3 protein. In the presence of the C-terminal part of the cofactor, the protease activity was enhanced [47]. 

### 2.2. Open and Closed Conformations of the Protease

**Figure 2:**
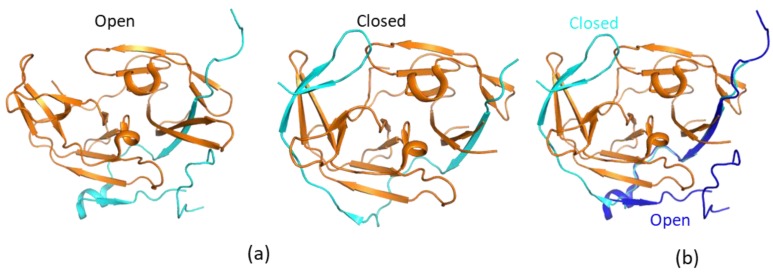
Open and closed conformations of the proteases. (**a**) Open and closed conformations of WNV protease. The non-structural protein 2B (NS2B) and NS3pro are shown in cyan and orange, respectively. (**b**) Structural comparison of open and closed proteases. The structures of open (PDB identifier (ID) 2FOM) and closed (PDB ID 2FP7) proteases are shown. The NS2B C-terminal portion from the cofactor is away from the active site to form an open conformation. NS2B in the open and closed conformations is shown in blue and cyan, respectively. To make it clear, only NS3 from the closed conformation is shown.

The structures of free WNV and DENV NS2B–NS3pro were first determined, and they suggested that the free protease exists in an open conformation which lacks protease activity [37,38,47]. Although the folding of NS3pro is almost the same as those in complexes with inhibitors, the C-terminal region of the NS2B cofactor positions away from the active site (Figure 2). This conformation is, therefore, indicated as the open conformation. This open conformation is inactive as the protease activity requires the presence of amino acids from the C-terminal section of NS2B cofactor region. These residues form a β-hairpin structure through molecular interactions with the substrate [48,49]. Therefore, several strategies were applied to develop protease inhibitors based on the available structures, namely, inhibitors derived from substrate sequences, small-molecule compounds binding to the active site, allosteric inhibitors to induce the open conformation, and inhibitors to stabilize the open conformation [50]. A structural study indicated that free ZIKV NS2B–NS3 protease exists in the closed conformation [33], and accumulated NMR studies demonstrated that the closed conformation of the protease should be used in structure-based drug design [51]. The open conformation observed in the X-ray studies might be due to crystal packing, while exchanges are indeed present at the binding interface of NS3pro and the C-terminal region of NS2B cofactor. 

## 3. Solution NMR Studies on Flavivirus Proteases

Although X-ray structures of flavivirus proteases provide detailed information to understand the interactions between protease and inhibitors, solution NMR spectroscopy provides complimentary information to better understand the structural information obtained using X-ray crystallography. Solution NMR spectroscopy was used to probe protease and inhibitor interactions. In addition, NMR studies suggested that the closed conformation should be used in structure-based drug design. 

### 3.1. Ligand-Bound Proteases Form Closed Conformation

Solution NMR studies on WNV NS2B–NS3pro protease to understand its structures were first carried out by Otting’s group. In one study, a modified protocol was developed to produce an isotopically labeled WNV protease for NMR studies [52]. The solution NMR spectra of WNV NS2B–NS3pro in complex with a small-molecule inhibitor were collected, and resonance assignment was obtained to analyze the solution structures of WNV protease. The study demonstrated that WNV protease adopted the closed conformation when it was bound to a small-molecule compound. This compound was proven to bind to the active site of the protease as nuclear Overhauser effects (NOEs) between protease and the compound were identified [52]. In a follow-up study, the ^1^H–^15^N-heteronuclear single quantum coherence (HSQC) spectra of WNV protease in the absence and presence of an inhibitor were collected and compared. Significant changes in the numbers of the detectable cross-peaks in the ^1^H–^15^N-HSQC spectra were observed when the protease formed a complex with an inhibitor [53]. 

**Figure 3:**
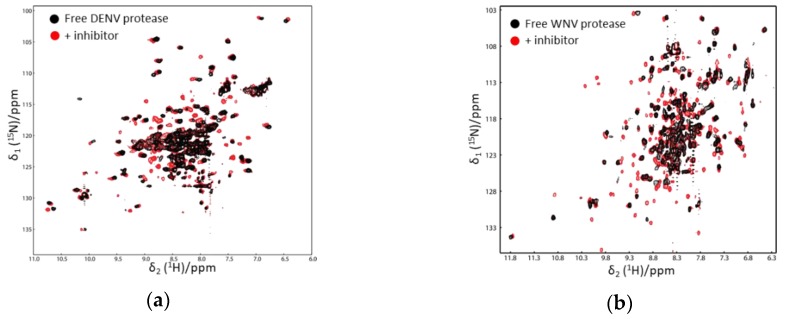
^1^H–^15^N-heteronuclear single quantum coherence (HSQC) spectra of Dengue virus (DENV) (**a**) and WNV protease (**b**) in the absence and presence of an inhibitor. The spectra of free and ligand-bound proteases are shown in black and red, respectively. More cross-peaks were observed when the protease formed a complex with an inhibitor. The spectra were obtained from Reference [58] with slight modifications. Similar results were also obtained in other studies [52,53].

In the ^1^H–^15^N-HSQC spectrum of free WNV protease, the cross-peaks corresponding to several residues at the active sites and from the C-terminus of the NS2B cofactor were not observed, which might be caused by the conformational exchanges of the C-terminal part of the cofactor region [53]. On the other hand, signals of residues from the N-terminal portion of the cofactor were observed in the ^1^H–^15^N-HSQC spectra of both free and ligand-bound proteases [53,54]. Such results demonstrate that the N-terminal portion of the factor forms a stable complex with NS3. The cross-peaks corresponding to the residues from the C-terminal part of the cofactor region and close to the active site of NS3pro were observed when the protease formed a complex with inhibitors. Similar results were found for DENV and ZIKV proteases [55,56,57]. The protease in complex with inhibitors/substrates exhibited well-dispersed cross-peaks in the ^1^H–^15^N-HSQC spectra [35,51,54,58,59,60] (Figure 3). Further chemical shift analysis demonstrated that the C-terminal region of NS2B cofactor region formed ordered structures in solution, and the protease was in the closed conformation [35,51,55,56,57]. Pseudocontact shift (PCS) was used to analyze a DENV protease construct in which a protease cleavage site was introduced. The result indicated that the protease adopted the closed conformation in solution [61]. A later study also showed that adding a protease cleavage site in the ZIKV protease construct resulted in a protease forming the closed conformation, in which the residues at the cleavage site had interactions with both NS3 and the C-terminus of NS2B cofactor [35]. 

### 3.2. Free NS2B–NS3pro Forms Closed Conformation in Solution 

To further probe the conformations of free protease in solution, a protease construct was labeled with paramagnetic tags. A paramagnetic relaxation enhancement (PRE) experiment and relaxation analysis demonstrated that free WNV protease was predominant in the closed conformation [61]. The C-terminal region of NS2B cofactor might exist in multiple conformations or dissociate from NS3, which contributes to signal broadening in the ^1^H–^15^N-HSQC spectrum of free protease. In the absence and presence of an inhibitor, the artificial linker used in the study is dynamic in solution. This linker was used for improving protease yield and purity without affecting enzymatic activities [19]. The DENV protease containing the same glycine-rich linker exhibited poorly dispersed cross-peaks in the ^1^H–^15^N-HSQC, making backbone resonance assignment challenging [62]. By tagging DENV protease with paramagnetic lanthanides, the protease was confirmed to contain the open conformation in its free form. The closed conformation can be readily induced by adding positively changed small-molecule inhibitors [62]. The conformational exchanges of DENV protease were clearly investigated using PRE experiments with different lanthanides. Using an ^19^F-NMR study, the free DENV protease with a glycine-rich linker was shown to contain both open and closed conformations in solution. Addition of a trypsin inhibitor (bovine pancreatic trypsin inhibitor (BPTI)) enabled reducing the population of the open conformation [63]. The conformation of DENV protease in solution was explored by NMR using an NS2B–NS3pro complex without any artificial linker. The protease complex in solution exhibited nicely dispersed cross-peaks in the ^1^H–^15^N-HSQC spectrum. Backbone assignment and a PRE experiment demonstrated that the free DENV protease existed in the closed conformation [20]. The artificial linker might affect the dynamics of NS2B to generate a spectrum with broadened signals. Indeed, comparison of the ^1^H–^15^N-HSQC spectra of ZIKV protease in the absence and presence of the glycine-rich linker revealed obvious chemical shift changes on a few residues, demonstrating that the linker is able to affect the chemical environment of some residues [57]. 

### 3.3. Free NS2B Cofactor Region and NS3pro Are Unstructured 

Free DENV NS3pro was not soluble and present in the inclusion bodies when it was expressed in *Escherichia coli*. It was able to be purified in the denatured form in the presence of 8 M urea. Purified NS3pro was able to be refolded into a buffer while the far-ultraviolet (UV) circular dichroism (CD) spectrum suggested that NS3pro alone contained approximately 29% extended strand and 4% helix. The narrow dispersion of the cross-peaks in the ^1^H–^15^N-HSQC spectrum suggests the lack of tertiary packing [64]. Backbone resonance assignment for the refolded NS3pro was achieved using heteronuclear NMR experiments, and chemical shift analysis of the Cα and Cβ atoms demonstrated that free NS3pro was not structured in solution [64]. In the presence of the cofactor region, NS3pro formed a well-folded structure which is similar to that derived from X-ray crystallography [20]. The structure of the free full-length NS2B in detergent micelles was investigated using solution NMR study [18]. It was observed that the transmembrane regions of full-length NS2B formed helical structures in solution, while the cofactor region was unstructured in the absence of NS3pro [18]. This cofactor region alone could be over-expressed and purified in *E. coli*. It was also soluble in water, and CD analysis suggested that it may contain some helical structures [64]. For the cofactor region from ZIKV NS2B, the free form was unstructured in solution [33]. Although the NMR studies on free cofactor region, full-length NS2B, and NS3pro did not provide atomic structures, these studies provided direct evidence to show the importance of the complex in regulating the function of viral protease. 

### 3.4. NMR in Guiding Protease Construct Design 

**Figure 4:**
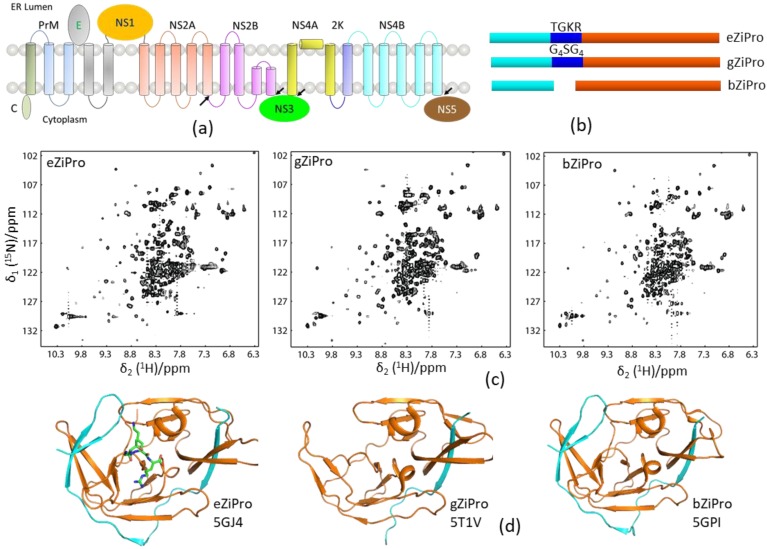
Solution NMR spectra of different protease constructs. (**a**) Diagram of the viral polypeptide. The arrows indicate the viral protease cleavage sites. (**b**) Current constructs used for ZIKV protease. The NS2B cofactor, linker, and NS3pro are shown in cyan, blue, and orange, respectively. (**c**) ^1^H–^15^N-HSQC spectra of the three protease constructs. (**d**) X-ray structures of these three constructs. The PDB access codes of structures are shown. The NS2B and NS3 are shown in cyan and orange, respectively. The enzyme cleavage site (TGKR) peptide in eZiPro is shown in sticks.

One-dimensional (1D) proton NMR can be used to monitor the folding of a purified protein. Constructs with high-quality X-ray structures usually generate high-quality NMR spectra [65]. NMR spectroscopy is, therefore, useful for evaluating the folding of the protease constructs which can be used for X-ray studies. As aforementioned, an unlinked DENV protease construct exhibited a better resolved ^1^H–^15^N-HSQC spectrum than the conventional DENV protease that contains a glycine-rich linker (G_4_SG_4_). In the structural studies of ZIKV protease, three constructs bZiPro, eZiPro, and gZiPro were designed (Figure 4). These constructs contained NS2B cofactor region and NS3pro without a linker, an enzyme cleavage site (TGKR), and a glycine-rich linker (G_4_SG_4_). All these constructs were able to be crystallized, and the studies showed that the C-terminal region of NS2B cofactor was structured in bZiPro and eZiPro and unstructured in gZiPro (Figure 4). NMR studies indicated that exchanges exist in bZiPro and gZiPro, while addition of substrate or inhibitors can suppress the exchanges to give rise to spectra in which the signals from residues at the NS2B–NS3 interface are detectable (Figure 4). The bZiPro construct was utilized in providing protein–ligand interactions because several crystal structures of protease complexes were solved [33,55,56,66]. Although all different constructs exhibited similar enzymatic activities, the unlinked protease complex without any artificial linker should be used in drug discovery. 

### 3.5. Other Applications 

As NS2B is a membrane protein, NS3 might also have molecular interactions with cell membranes. A hydrophobic loop from DENV NS3 conserved among flaviviruses was suggested to bind to the cell membrane [67]. NMR was then used to investigate molecular interactions between WNV protease and membranes. Using a titration experiment, several residues were identified to bind to detergent micelles which were used as a mimic of the cell membrane [68]. Such a study provided direct evidence to demonstrate the specific interactions between NS3 and the cell membrane. 

In addition, flaviviral proteases were used as a model system for developing novel NMR methods for structure determination and ligand binding studies [69,70,71]. Distance restraints for structural determination are usually obtained from NEOSY spectra, which is challenging for some proteins due to signal overlaps or conformational exchanges. PCS data from DENV protease were generated by tagging lanthanide to the target protein [72]. The resulting data can be used as distance restraints or used as an input for generating computational models. The structures of NS2B can be obtained using a combination of PCS and computational model using Rosetta [70]. Such a strategy is an efficient and reliable way for determining structures of proteins [73]. 

Using ZIKV protease as one of the model proteins, ^1^H-NMR was shown to be able to probe protein–ligand interactions. Although ^1^H-NMR is very sensitive, the overlaps between protein and ligand signals hinders its application in probing protein and ligand interactions. In a recent study, a trimethylsilyl (TMS) tag was developed to solve this problem, as TMS exhibits a chemical shift near 0 ppm in ^1^H-NMR spectra. A cysteine residue is required in a protein for the tagging reaction. As ZIKV protease harbors enzymatic activity, the impact of tag modification on the activity of the target protein can be analyzed. It was shown that the TMS tags were able to react with a cysteine residue introduced into ZIKV protease without affecting enzymatic activity. The resulting protease was able to be utilized for probing protein–ligand interactions. In addition, ^1^H-NMR can also be used to estimate the dissociation constant for the interactions in the slow exchange regime [71]. 

### 3.6. Summary 

NMR studies provide information to understand structures, conformational exchanges, and dynamics of these proteases. Introducing a linker in the protease is a great strategy in recombinant protein production, while chemical environment of some residues could be affected even though the linker is unstructured. Together with X-ray crystallography and computational methods, NMR provides a meaningful view of protein structures. 

## 4. Roles of NMR in Antiviral Development 

High-resolution structures of quite a few flavivirus proteases were obtained using X-ray crystallography, making structure-based drug design feasible. In addition, computer-based ligand screening can be carried out readily to screen potential inhibitors. Although it is challenging to obtain solution NMR structures of protease in complex with inhibitors as it is time-consuming, quite a few NMR experiments were carried out to understand protein structures and ligand interactions (Table 1). NMR will play important roles in drug development as it can be utilized to identify and confirm compounds which have a wide range binding affinities (mM to nM) to the protease. 

### 4.1. NMR in Developing Small-Molecule Inhibitors

NMR is used to probe protease and small-molecule interactions to confirm the hits identified using other methods such as high-throughput screening (HTS) [74]. NMR is a well-known method to evaluate compounds with different binding affinities to a target. This method was routinely used to evaluate compounds derived from HTS. The dissociate constant can be obtained for compounds whose exchanges are fast on the NMR time scale [75]. The NOEs between protease and inhibitors were used to determine the binding mode, leading to comprehensive understanding of the complex in solution [52]. Together with mass spectrometry and X-ray crystallography, NMR was also used to monitor the molecular interactions between ZIKV protease and an irreversible inhibitor [55]. In addition, NMR was successfully applied to probe the interactions between protease and inhibitors with weak binding affinities. Fragments identified using thermal shift assay were confirmed to bind to the active site of ZIKV protease. Comparison of the ^1^H–^15^N-HSQC spectra of ZIKV protease in the absence and presence of the fragments showed that fragment binding did not suppress the exchanges in the protease, which is different from other potent inhibitors [33,66]. Such binding studies provide compensative information to the X-ray structures. 

### 4.2. NMR in Developing Peptidic Inhibitors 

**Figure 5:**
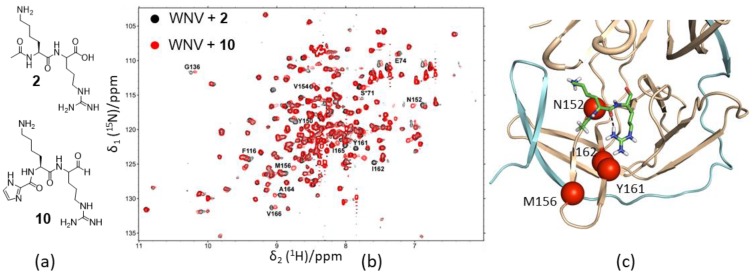
Using NMR spectroscopy to understand the structure-activity relationship (SAR) of peptidic inhibitors. (**a**) Structures of inhibitors used for comparison. (**b**) ^1^H–^15^N-HSQC spectra of WNV protease bound to compound **2** (black) and **10** (red). The residues which exhibited chemical shift difference are labeled with residue name and sequence number. (**c**) A model of WNV protease in complex with compound **2**. The residues which exhibited chemical shift changes more than 0.1 ppm are shown as red spheres and labeled with residue name and sequence number. The figures were obtained from Reference [54] with permission.

As the active site of flavivirus proteases is charged, it is challenging to develop small-molecule inhibitors [21,46]. Peptides derived from substrates could be further developed into drugs. NMR was used to evaluate the effect of peptide length or addition of a warhead on the protease binding. Although the dissociation constant was not obtained in the study, NMR experiments provided detailed information to understand the structure–activity relationship (SAR) of peptidic inhibitors against WNV protease [54]. Firstly, obvious chemical shift perturbation was observed when the peptide was mixed with WNV protease. Chemical shift analysis indicated that the protease formed the closed conformation in solution. Secondly, filtered-NOESY experiments showed that residues including F130 and I155 were important for inhibitor binding. Lastly, although quite a few new cross-peaks appeared in the ^1^H–^15^N-HSQC spectrum of the WNV–inhibitor complex, analyzing the difference of the chemical shift changes induced by different inhibitors was carried out to understand the SAR. The result provided clear information to understand the roles of various chemical groups from the inhibitors in protease binding and residues from the protease critical for inhibitor binding (Figure 5). NMR spectroscopy could readily show the importance of P1-Arg residue for the activity of the inhibitor, as modifying its side chain abolished its interaction with WNV protease. In the study of ZIKV protease, NMR spectroscopy was used to determine the dissociation constants of peptides with different lengths [33,76]. The dynamics of the peptide in the complex can also be explored, which is critical in inhibitor development. It should be noted that there are numerous inhibitors derived from substrates, which might be challenging for developing peptidic inhibitors against flavivirus proteases. This is due to the charged surface of the protease, which makes modifying the side chains of peptides challenging [46]. 

### 4.3. Fragment-Based Drug Design Using NMR Spectroscopy 

Fragment-based drug design (FBDD) is widely used in target-based drug discovery [78]. It was proven to be a powerful tool to develop potent inhibitors targeting diverse targets including undruggable targets [79,80]. FBDD was utilized to identify hits binding to flavivirus proteases. Using saturation transfer difference (STD) NMR experiments, quite a few fragments were identified. Using an STD-based competition assay, compounds were shown to bind to different sites of WNV protease. The inhibitor binding site was also confirmed using ^1^H–^15^N-HSQC experiments. These identified hits can serve as a starting point for optimization [81]. In another study, over 20 fragments were identified which were able to improve the thermal stability of ZIKV protease. X-ray crystal studies demonstrated that the fragments were bound to P1 position of the active site. STD NMR confirmed the binding in solution, and further ^1^H–^15^N-HSQC experiments demonstrated that fragment binding to ZIKV protease did not abolish the exchanges in the protease [33,66]. 

### 4.4. Other Inhibitors 

The trypsin inhibitor BPTI was able to inhibit enzymatic activities of DENV, WNV, and ZIKV protease. Structural studies showed that BPTI-bound protease adopted the closed conformation in solution [82]. BPTI was used as a tool compound for structural and biochemical studies of flavivirus proteases. Solution NMR studies also demonstrated that DENV protease was in the closed conformation when it formed a complex with BPTI [51]. 

In view of the characteristics of proteases, an effort was made to develop allosteric inhibitors. Stabilizing the open conformation of the protease might be a mechanism of allosteric inhibitors. Ala125 of DENV protease was proposed to be a binding site for this type of inhibitors [50]. Nonstandard macrocyclic peptides were proven to be non-competitive inhibitors of ZIKV protease [83]. A small-molecule inhibitor was able to inhibit DENV protease through an allosteric mechanism. The crystal structure showed the mechanism of action of the inhibitor which has a great potential to be developed into antivirals [84]. No NMR studies were carried out to understand the molecular interactions between protease and these allosteric inhibitors. The protease construct with a glycine-rich linker might be useful in evaluating the allosteric inhibitors as it contains multiple conformations [85]. ^19^F-NMR will play important roles in evaluating such inhibitors, as it can provide a fast and definitive measurement to understand conformational changes induced by a ligand [63,86]. 

### 4.5. Summary 

Besides understanding the SAR of compounds, NMR is particularly important in evaluating the developed protease inhibitors with different binding affinities. It can also play a role in FBDD, as well as confirm hits identified using other screening methods. The aforementioned constructs (Figure 4) can be utilized in evaluating different types of inhibitors such as allosteric inhibitors. 

## 5. Conclusions

Numerous NMR studies on proteases from DENV, WNV, and ZIKV were carried out, which provide insights into conformational exchanges and dynamics. NMR spectroscopy was able to test the folding of different protease constructs which could be used in evaluating different types of inhibitors. This technique can play important roles in hit identification and confirmation steps, especially in FBDD. Together with X-ray crystallography, the mechanism of action for an inhibitor can be well understood. It should also be noted that NMR plays important roles in structural studies of viral proteins, as several viral proteins are challenging to be crystallized. 

## Figures and Tables

**Table 1:** NMR experiments used for flavivirus proteases. PCS—pseudocontact shift; 3D—three-dimensional; 1D—one-dimensional; PRE—paramagnetic relaxation enhancement.

Experiment	Applications	References
^1^H–^15^N-HSQC^1^	Protein folding, protein–ligand interactions	[54,58]
^1^H–^15^N-HSQC	For amino acid-specific labeled samples	[53]
PCS	Pseudocontact shift	[61,70]
3D experiments	Backbone resonance assignment	[57,76]
^19^F-NMR	Protein conformational changes and ligand binding	[63]
NOESY	Protein–ligand interactions	[52]
1D ^1^H-NMR	Protein–ligand interactions	[71]
Filtered NOESY	Determining ligand-binding sites	[52,54]
Relaxation	Protein dynamics	[33,57]
PRE	MTSL, protein structure determination	[20,33]
PRE	Protein structure determination using lanthanide tag	[62,69,77]
STD NMR	Ligand binding, hit identification in FBDD	[66,75]

^1^ This type of experiment is frequently used for both mapping the ligand binding site and confirming the conformation of the protease.

## Abbreviations

NMR	Nuclear magnetic resonance
HSQC	Heteronuclear single quantum coherence
DENV	Dengue virus
WNV	West Nile virus
ZIKV	Zika virus
BPTI	Bovine pancreatic trypsin inhibitor
PRE	Paramagnetic relaxation enhancement
NOE	Nuclear Overhauser effect
PCS	Pseudocontact shift
UV	Ultraviolet
CD	Circular dichroism
1D	One-dimensional
STD	Saturation transfer difference
HTS	High-throughput screening
MTSL	1-Oxyl-2,2,5,5-tetramethyl-Δ3-pyrroline-3-methylmethanethiosulfonate
FBDD	Fragment-based drug design
